# A Tissue Engineered 3D Model of Cancer Cell Invasion for Human Head and Neck Squamous-Cell Carcinoma

**DOI:** 10.3390/cimb46050250

**Published:** 2024-04-28

**Authors:** Manuel Stöth, Anna Teresa Mineif, Fabian Sauer, Till Jasper Meyer, Flurin Mueller-Diesing, Lukas Haug, Agmal Scherzad, Maria Steinke, Angela Rossi, Stephan Hackenberg

**Affiliations:** 1 Department of Otorhinolaryngology, Plastic, Aesthetic and Reconstructive Head and Neck Surgery, University Hospital Würzburg, 97080 Würzburg, Germany; stoeth_m@ukw.de (M.S.); meyer_t2@ukw.de (T.J.M.); muellerdie_f@ukw.de (F.M.-D.); scherzad_a@ukw.de (A.S.); maria.steinke@isc.fraunhofer.de (M.S.); 2 Chair of Tissue Engineering and Regenerative Medicine, University Hospital Würzburg, 97070 Würzburg, Germany; anna@mineif-online.de (A.T.M.);; 3 Institute of Pathology, University of Würzburg, 97080 Würzburg, Germany; lukas.haug@uni-wuerzburg.de; 4 Fraunhofer Institute for Silicate Research ISC, 97082 Würzburg, Germany; angela.rossi@isc.fraunhofer.de

**Keywords:** head and neck squamous-cell carcinoma, tissue engineering, oral mucosa, 3D tissue model

## Abstract

Head and neck squamous-cell carcinoma (HNSCC) is associated with aggressive local invasiveness, being a main reason for its poor prognosis. The exact mechanisms underlying the strong invasive abilities of HNSCC remain to be elucidated. Therefore, there is a need for in vitro models to study the interplay between cancer cells and normal adjacent tissue at the invasive tumor front. To generate oral mucosa tissue models (OMM), primary keratinocytes and fibroblasts from human oral mucosa were isolated and seeded onto a biological scaffold derived from porcine small intestinal submucosa with preserved mucosa. Thereafter, we tested different methods (single tumor cells, tumor cell spots, spheroids) to integrate the human cancer cell line FaDu to generate an invasive three-dimensional model of HNSCC. All models were subjected to morphological analysis by histology and immunohistochemistry. We successfully built OMM tissue models with high in vivo–in vitro correlation. The integration of FaDu cell spots and spheroids into the OMM failed. However, with the integration of single FaDu cells into the OMM, invasive tumor cell clusters developed. Between segments of regular epithelial differentiation of the OMM, these clusters showed a basal membrane penetration and lamina propria infiltration. Primary human fibroblasts and keratinocytes seeded onto a porcine carrier structure are suitable to build an OMM. The HNSCC model with integrated FaDu cells could enable subsequent investigations into cancer cell invasiveness.

## 1. Introduction

Head and neck squamous-cell carcinoma (HNSCC) occurs in the cell lining of the oral cavity, pharynx, and larynx. According to estimates from the Global Cancer Observatory (GLOBOCAN), HNSCC is the sixth most prevalent cancer globally, with approximately 890,000 new cases and 450,000 deaths each year, posing a significant public health challenge [1]. Major risk factors for HNSCC include tobacco and alcohol consumption [2], as well as human papillomavirus (HPV) and Ebstein–Barr virus (EBV) infection. Betel quid chewing [3], poor oral hygiene [4], and chronic traumatism [5] are additional factors that contribute to an increased risk of developing this cancer. Treatment for HNSCC primarily involves a combination of surgery, radiotherapy, and chemotherapy, based on the tumor stage and pathological diagnosis. Further treatment options include immunotherapy and targeted therapy. The local recurrence rate of HNSCC varies widely, depending on several factors such as the location of the tumor, the stage at diagnosis, and the treatment methods used. Despite the use of multimodal therapy, the local–regional recurrence rate for advanced HNSCC can be as high as 50% [6]. Notably, recurrent and metastatic disease emerges as a major cause of mortality in HNSCC [7]. Despite remarkable advances in surgery, radiation, and medical therapy over the past few decades, the prognosis for HNSCC remains poor, with a 5-year overall survival rate ranging from 25% for hypopharyngeal cancer to 59% for laryngeal cancer [8].

An in-depth understanding of HNSCC biology is critical to improve the prognosis. One challenge in achieving this is the development of suitable cancer models. So far, several in vitro cancer studies have been carried out using two-dimensional (2D) monolayer cell cultures. Although such models are easy to handle and reproducible, they fail to include the complex interplay between cancer cells, non-malignant cells, and acellular components within the tumor microenvironment (TME) [9]. For example, gene expression differs between 2D monolayer culture and three-dimensional (3D) cancer models, emphasizing the role of cell–cell interaction in cancer [10]. Furthermore, 3D cancer models have been shown to be more resistant to anticancer drugs compared to 2D models [11]. In addition, there is compelling evidence highlighting the importance of non-malignant cells in tumor progression [12]. Additionally, acellular components, including molecules of the extracellular matrix (ECM), have been shown to influence the phenotype of cancer cells [13]. Organoids represent an advanced 3D culture technology to mimic the complex physiology within human organs and tumors. However, organoids fail to integrate the whole TME [14] and have limitations in terms of studying cancer cell invasiveness and metastasis [15]. Conventional and patient-derived xenograft animal models are acknowledged as sophisticated tools for cancer research. They allow for detailed study of cancer metastasis [16] and the TME [17,18], and enable in vivo drug testing [19,20,21]. However, their informative value is also limited: in addition to ethical issues, costs, and availability [22], there are genetic [23], immunological [24], and stromal [25] differences between human, grafted, and experimental animal tumors. All the mentioned models make valuable contributions to preclinical cancer research. Nevertheless, all platforms have specific disadvantages that limit their use as cancer models. Thus, there is an unmet need for further cancer models to study HNSCC.

Compared to 2D in vitro models, tissue-engineered 3D models represent a complex culture format more closely resembling the physiological conditions of human tumors [26]. With the integration of TME components, cancer cells can be subjected to cellular, stromal, and biophysical stimuli. A suitable cell carrier is crucial for the generation of such 3D tissue models. This carrier represents a scaffold of ECM, which ensures the 3D structure of the model and allows cell-to-ECM interactions. A distinction is made between natural and synthetic cell carriers [27]. Advantages of natural cell carriers include low antigenicity [27], abundance in structural and functional proteins, and the fact that they allow for better epithelial cell attachment compared to synthetic cell carriers [28]. An example of a natural scaffold is the acellular carrier structure of porcine small intestinal submucosa with preserved mucosa (SIS/MUC), which consists of cross-linked collagen and elastin fibers [29]. It has previously been used to study the drug responses of tumor cell lines in human 2D and 3D lung cancer models [30]. In addition to providing a biologically intact ECM, SIS-based scaffolds enable fibroblast migration, epithelial differentiation, and the formation of a basement membrane [31]. The latter is essential for generating an HNSCC model adequately able to study tumor cell invasiveness—a major driver of tumor metastasis and recurrence.

Such a model could provide an ideal basis to elucidate specific interactions within the TME and to gain more insights into the interplay between cancer cells and normal adjacent tissue at the invasive tumor front. Therefore, the aim of this study was to generate a 3D tissue model of invasive HNSCC in which tumor cells are integrated into a healthy oral mucosal model (OMM) consisting of epithelium, basement membrane, and connective tissue.

## 2. Materials and Methods

Patients and specimens: This study was conducted according to the guidelines of the Declaration of Helsinki and was approved by the local ethic committee (Institutional review board number: 182/10). After written informed consent was obtained from each subject, fresh oral mucosa was obtained exclusively from donors with intact oral mucosa who underwent elective surgery (e.g., oral surgical repositioning osteotomies, trauma surgery, or metal removal). Exclusion criteria included a history of radiotherapy to the head and neck, inflammatory or malignant oral diseases, smoking, and high-risk alcohol consumption. Samples were collected from eight participants aged between 18 and 73 years. 

Primary cell isolation: Following intraoperative collection, oral mucosa tissue was cut into 2 mm slices and then incubated in dispase (Life-Technologies, Carlsbad, CA, USA) at 4 °C for 16 h. Thereafter, the epithelium and lamina propria were detached from one another. The epithelium was then processed into a single-cell suspension of keratinocytes by mechanical and brief enzymatic disaggregation with trypsin (Life-Technologies). Thereafter, keratinocytes were transferred into a culture flask. The remaining tissue pieces consisting of lamina propria were placed into a T25 cell culture flask, allowing fibroblasts to spread out of the tissue and further proliferate. Once keratinocytes and fibroblasts reached confluency, they were cryopreserved until further usage.

Cell culture: Fibroblasts were grown in DMEM (Thermo Fisher Scientific, Waltham, MA, USA) supplemented with 10% fetal calf serum (FCS) (Bio&Sell, Feucht, Germany) and keratinocytes in E1-Medium consisting of EpiLife^®^Medium (Life Technologies) supplemented with 60 μM calcium chloride, 5 mL human keratinocyte growth supplement (Life Technologies), 0.2% bovine pituitary extract, 1 μg/mL insuline-like growth factor-I, 0.18 μg/mL hydrocortison, 5 μg/mL transferrin, and 0.2 ng/mL epidermal growth factor. For co-culture experiments under submerged and air–liquid interface (ALI) culture, E2 and E3-Media were used. E2-Medium consisted of E1-Medium supplemented with 2.4 mL 300 mM calcium chloride, and E3-Medium was further supplemented with 500 μL keratinocyte growth factor (Sigma Aldrich, St. Louis, MS, USA) and 500 μL ascorbyl phosphate. The hypopharyngeal squamous cell cancer cell line FaDu [32] was obtained from the American Type Culture Collection. Cells were grown in RPMI (Life Technologies) supplemented with 10% FCS. All cell culture media contained 0.1 mg/mL streptomycin and 100 U/mL penicillin (Life Technologies). All cells were tested for mycoplasma using the Venor^®^GeM Classic Kit (Minerva Biolabs, Berlin, Germany) every 4 weeks to ensure that they were free of contamination. For the generation of multicellular tumor spheroids, 50 μL of 0.5% agarose was added to each well of a 96-well plate and incubated overnight at 4 °C. 5000 FaDu cells in 100 μL were seeded into each well and incubated for 4 d. For microscopic detection, FaDu cells were transduced lentivirally to constitutively express red fluorescent protein (RFP). Therefore, FaDu cells were incubated overnight with RFP lentivirus and polybrene (Sigma Aldrich). Next, 2 mL of cell-specific medium was added. On the third day, the medium along with viral particles was changed and rinsed, and the culture was continued until the twenty-sixth day. Next, selection of RFP expressing cells was performed by adding 0.25 μg/mL puromycin (InvivoGen, Toulouse, France), increasing the dose to 0.5 μg/mL on day 31 and 1 μg/mL on day 36. Successful transduction was confirmed by fluorescence microscopy with the detection of more than 90% RFP-expressing FaDu cells.

**Figure 1:**
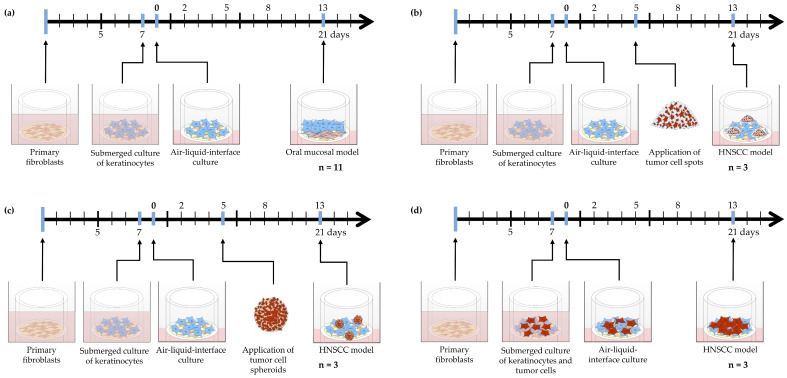
(**a**) Experimental design used to generate an oral mucosal model (OMM) and (**b**–**d**) different methods of integrating FaDu tumor cells into the OMM. (**b**) Integration of multicellular tumor cell spots. (**c**) Integration of multicellular tumor spheroids. (**d**) Integration of single tumor cells. Oral mucosal model (OMM), head and neck squamous-cell carcinoma (HNSCC).

Three-dimensional human oral mucosal model: Animal experiments were performed after previous approval by the ethical committee of the local government (approval number: 55.2-2532-2-256). All procedures conformed to the guidelines from Directive 2010/63/EU of the European Parliament on the protection of animals used for scientific purposes. After obtaining porcine small intestine as the carrier structure for our HNSCC model, the scaffold was prepared according to methods outlined in prior studies [29]. As a 3D scaffold for tissue model generation, decellularized porcine SIS/MUC was used, as previously described [31]. Briefly, pieces of the scaffold were mounted into cell crowns 13 mm in diameter. After preincubation, 50,000 fibroblasts were seeded in E1-Medium from the apical side to the cell crown. After incubation for seven days, 25,000 keratinocytes from the same donor were seeded on the apical side as well. Fibroblasts were used at passage 4, and keratinocytes at passage 2. The cells were cultured under submerged conditions with E2-Medium for 24 h, and subsequently as an ALI culture with E3-Medium for 12 d (Figure 1a). Eleven OMMs from eight different donors were generated. For each model, three technical replicates were generated.

Three-dimensional human head and neck squamous-cell carcinoma model: FaDu tumor cells were integrated into the human OMM in three different ways (Figure 1b–d). (i) Integration of multicellular tumor cell spots: On day 5 of ALI cultivation, medium was removed and 10,000 FaDu cells were seeded in 2 μL of RPMI medium from the apical side onto the OMM. After adding E3 media, ALI culture was continued for 9 more days. (ii) Integration of multicellular tumor spheroids: On day 5 of ALI cultivation, medium was removed and tumor cell spheroids were placed in the center of the apical side of the OMM. The culture was continued in the same way as in (i). For tumor cell detection, RFP-expressing FaDu cells were used. (iii) Integration of single tumor cells: On day 8 after incubation of fibroblasts within the SIS/MUC, a mixed suspension consisting of different ratios of FaDu cells and keratinocytes were seeded on the apical side into the cell crowns. For each method, three HNSCC models from two different donors were built. For each model, three technical replicates were generated. As in the OMM, the cells were cultured under submerged conditions with E2 medium for 24 h, and subsequently as an ALI culture with E3 Medium for 14 d. 

Histology and immunohistochemistry: Immunohistochemistry (IHC) was performed as previously described [33]. Briefly, samples were fixed in 4% formalin for 3 h, then embedded in paraffin and sectioned at 5 μm thickness using a Leica SM2010 R Sliding Microtome (Leica, Wetzlar, Germany). Following antigen retrieval using sodium citrate buffer and blocking of endogenous peroxidases using 3% H_2_O_2_, primary antibodies were diluted with antibody dilution buffer (DCS, Hamburg, Germany) in the mixing ratio specified below, added following the provider’s instructions, and incubated for 60 min at room temperature: Cytokeratin (CK) 10 1:100 (M7002, DAKO, Eching, Germany), CK14 1:2000 (HPA023040, Sigma-Aldrich, St. Louis, MO, USA), Ki67 1:100 (ab16667, Abcam, Cambridge, MA, USA), Collagen IV 1:500 (ab6586, Abcam), p53 1:50 (M7001, DAKO), and Vimentin 1:2000 (ab92547, Abcam). Primary antibody detection, signal enhancing, and chromogenic visualization were performed using the DCS Super Vision 2 HRP-Polymer-Kit (DAKO) according to the provider’s instructions. Negative controls were utilized for each experiment by omission of primary antibodies. Mayer’s hemalum solution (Merck, Darmstadt, Germany) was used for nuclear counterstain. H&E staining was performed using the following protocol. Tissue sections were stained for cell nuclei with Mayer’s hemalum solution, followed by a ten-minute flush with tap water. Then, the sections were stained with eosin for three minutes and subsequently rinsed under running tap water for 30 s to eliminate excess dye. After staining, the tissue underwent dehydration through an ascending series of alcohol concentrations, followed by immersion in xylene. Sister slides were used for all histology and IHC staining. Images were acquired using the BZ-9000 BIOREVO System (Keyence, Neu-Isenburg, Germany) and processed with BZ-II Analyzer and BZ-II Viewer software (Version 2.1, Keyence). Figures were illustrated with Microsoft Paint (Redmond, WA, USA) and compiled with Microsoft PowerPoint (Version 2312).

## 3. Results

Generation of a reproducible 3D human oral mucosal model: The first step in establishing a human HNSCC model was the generation of a healthy OMM, which resembled the human oral mucosa. The aim was to evaluate the suitability of primary fibroblasts and keratinocytes from the oral cavity to generate such OMM. The growth pattern and properties of the OMM were assessed via H&E staining and IHC. A decellularized porcine SIS/MUC was used for the generation of the OMM. SIS-based scaffolds have already proved to be a suitable basis on which to build human cancer tissue models [30,31,34]. 

**Figure 2:**
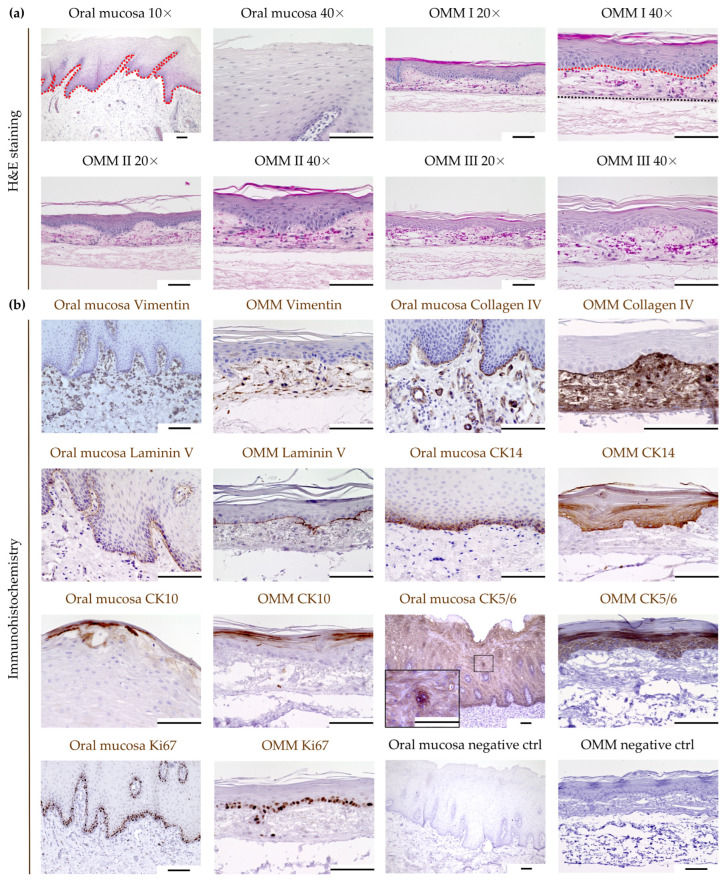
Generation of a 3D bioequivalent oral mucosa model (OMM). (**a**) Comparison of human oral mucosa with the OMM by H&E staining. The red dotted line separates the stratified squamous epithelium from the underlying lamina propria. The black dotted line separates the lamina propria from residual SIS/MUC. (**b**) Comparison of human oral mucosa with the OMM by immunohistochemistry. Scale bars represent 100 μm. Oral mucosa model (OMM), cytokeratin (CK), control (ctrl).

In all models, H&E staining showed a regular formed, keratinized, stratified squamous epithelium without any histological features of dysplasia. The epithelium of the OMM consisted of 10–15 cell layers with a thickness of around 100–120 μm and a regular stratification (Figure 2a). The thickness of the epithelium and lamina propria was comparable between different donors. However, in comparison to oral mucosa, the OMM exhibited a lower mucosal thickness. The overall thickness of the model, including the scaffold, was around 350 μm, with a diameter of 13 mm. IHC allowed us to further analyze the morphology and cell composition of the OMM (Figure 2b). In both the OMM and in oral mucosa, Vimentin-positive fibroblasts were exclusively found within the lamina propria, and collagen IV was expressed within the basal membrane and lamina propria. Furthermore, a continuously formed basal membrane was clearly identifiable by more intense collagen IV staining. Laminin V stained the basal membrane in oral mucosa. An identical Laminin V staining pattern was detectable in the OMM, providing further evidence for the formation of a basal membrane in the OMM. CK14 is a marker for keratinocytes of the basal layer [35]. Compared to oral mucosa, CK14 staining was more intense in OMM, but also decreased towards the apical surface. As in oral mucosa, suprabasal cells in the OMM stained positive for CK10. A matching staining was found in the OMM. All layers of oral mucosa stained moderately positive for CK5/6. Similarly, all layers of the OMM were positive for CK5/6, though with more intense staining. Proliferating Ki67 cells were found in the basal epithelial layer and occasionally within the lamina propria.

Overall, 11 OMM from eight different donors were generated and evaluated. The success in establishing the OMM indicates the suitability of primary oral fibroblasts and keratinocytes. Furthermore, the comparable properties between each OMM indicate the reproducibility of the model.

**Figure 3:**
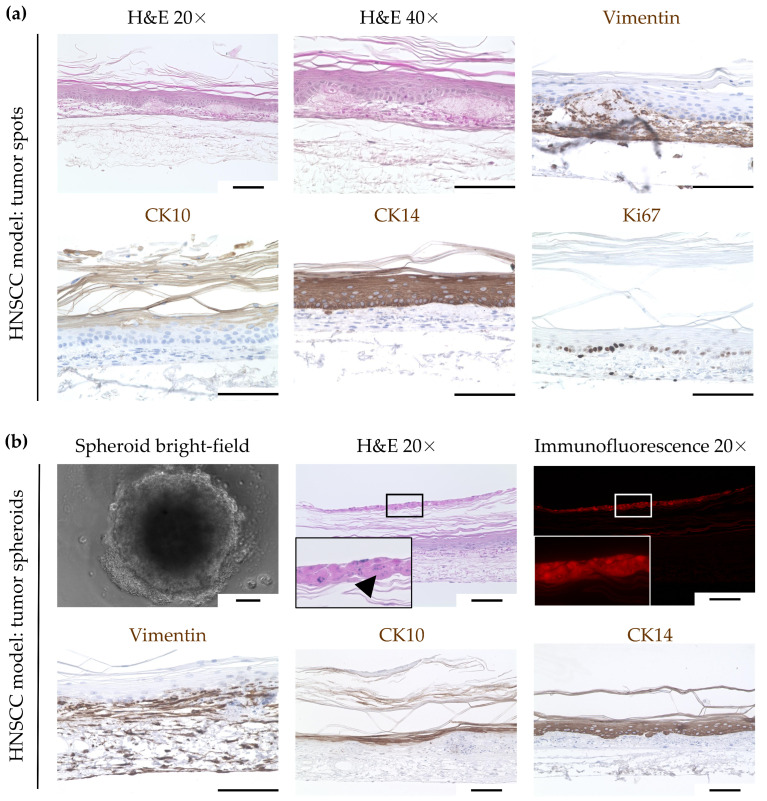
Failed integration of (**a**) FaDu tumor cell spots (**b**) and tumor spheroids into the oral mucosa model (OMM). FaDu cells were transduced lentivirally to constitutively express red fluorescent protein (RFP). Analysis by H&E staining and immunohistochemistry showed a keratinized stratified squamous epithelium, similar to the OMM. While, in (**a**), no tumor cells could be detected, in (**b**), RFP-positive tumor cells appeared on the stratum corneum as remnants of the spheroid. The arrowhead indicates tumor cells atop the stratum corneum, which are strongly eosinophilic with fragmented nuclei. Scale bars represent 100 μm. Head and neck squamous-cell carcinoma (HNSCC), cytokeratin (CK), red fluorescent protein (RFP).

Failed integration of multicellular tumor cell spots and tumor spheroids into the 3D human oral mucosal model: Next, we aimed to use the OMM as a basis to generate human HNSCC models. Therefore, we tested different ways of integrating FaDu hypopharyngeal squamous cell carcinoma cells into the OMM. One way was the seeding of FaDu tumor cell spots onto the OMM (Figure 1b). The histological analysis of H&E staining revealed a regular formed, keratinized, stratified squamous epithelium, as seen in the OMM. There were no signs of either malignancy or tumor cells. A further characterization was performed by IHC, which revealed a morphology similar to the OMM (Figure 3a).

Another method was the seeding of multicellular tumor spheroids (Figure 3b) onto the OMM on day 5 of ALI culture (Figure 1c). Histological analysis after nine more days showed tumor cells located on top of the stratum corneum, as seen with H&E staining and RFP-positive cells. The tumor cells were strongly eosinophilic with fragmented nuclei, possibly indicating apoptotic cells. All epithelial layers below, as well as the lamina propria, had regular morphology without any signs of malignancy, as seen in the OMM. This was further supported by IHC, showing a similar morphology compared to the OMM (Figure 3b).

**Figure 4:**
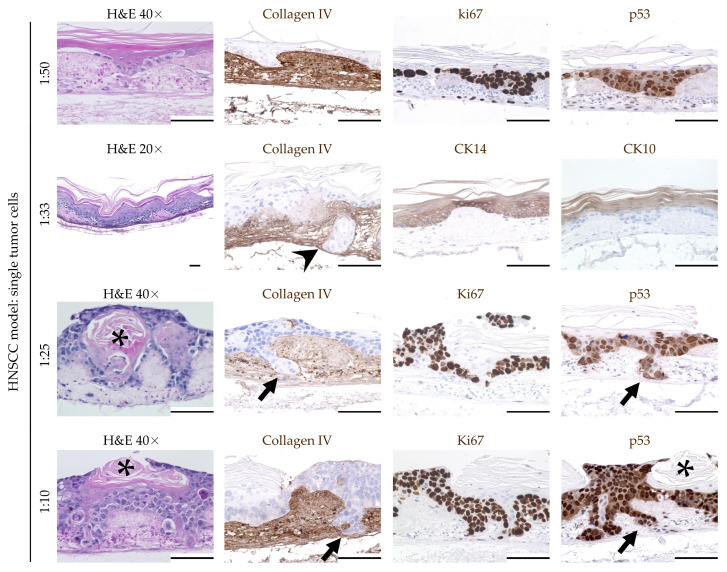
Integration of single tumor cells as a mixed suspension consisting of FaDu cells and keratinocytes. Depending on the mixing ratio, sections of normal stratification could still be detected at ratios up to 1:33. This was no longer the case at a ratio of 1:25. From a ratio of 1:33, there were clear signs of invasive tumor growth with basement membrane penetration and invasion into the lamina propria (arrowhead and arrows). Horn beads (asterisk), as another sign of malignancy, could be seen at ratios of 1:25 and 1:10. Scale bars represent 100 μm. Head and neck squamous-cell carcinoma (HNSCC), cytokeratin (CK).

Generation of a human head and neck squamous-cell carcinoma model through early integration of single tumor cells: Another way to transform the OMM into a human HNSCC model was the early integration of single tumor cells as a mixed suspension consisting of FaDu cells and keratinocytes (Figure 1d). For this purpose, different suspension ratios were seeded into the cell crowns eight days after the incubation of fibroblasts into the SIS/MUC. After a further 13 days in culture, the histological analysis revealed remarkable morphological differences between the cell ratios utilized compared to the previous models (Figure 4). 

When using a tumor cell-to-keratinocyte ratio of 1:50, we could still observe signs of stratification, as supported by regular upper cell layers and a continuously formed stratum corneum. The cells within the basal layers appeared to be irregular, with a nuclear pleomorphism and differences in size, indicating the formation of tumor cell clusters. This observation was further supported by local accumulations of Ki67- and p53-positive cells within these clusters. However, a penetration of the basal membrane with invasion into the lamina propria was not detected.

In contrast, at a ratio of 1:33, some segments of the model showed a disturbed epithelial architecture and invasive tumor growth. The arrowhead in Figure 4 highlights a segment of penetration of the basal membrane with invasion into the lamina propria. However, other segments of the model showed regular architecture, with organized stratification and a continuously formed stratum corneum. Basal membrane penetration was also detected at the ratios of 1:25 and 1:10. The arrows in Figure 4 show examples of tumor cell clusters invading into the lamina propria. In addition, most of the epithelial cells were positive for the proliferation marker Ki67 and the tumor suppressor p53. A regular epithelial architecture and an equal distribution of fibroblasts were no longer detectable. Instead, atypical cornification was found (Figure 4, asterisk).

In summary, integrating multicellular tumor spots and tumor spheroids into the OMM on day 5 of ALI culture failed. In contrast, early seeding of a mixture of keratinocytes and FaDu during the formation of the OMM enabled the establishment of a human HNSCC model. Depending on the ratio of keratinocytes and FaDu tumor cells, penetration of the basal membrane as well as a disrupted epithelial architecture could be detected. Table 1 provides a comparison of the results obtained from the different seeding approaches.

## 4. Discussion

The aim of the present study was to generate a 3D model of HNSCC that mimics the crosstalk at the invasive tumor front of human HNSCC by enabling cellular and stromal interactions. Our model is based on invasive cancer cells within an OMM which was established from primary human cells seeded on a porcine SIS/MUC. The focus of the study was set on morphological analysis based on histology and IHC. In summary, we showed that (1) an OMM could be generated from primary human fibroblasts and keratinocytes seeded onto a porcine SIS/MUC; (2) this OMM was reproducible, showed a regular pattern of differentiation, and therefore shared relevant properties with oral mucosa; and (3) HNSCC cells could be integrated into this model, which ultimately led to the generation of a 3D HNSCC model with features such as basal membrane penetration and cancer cell invasion into the lamina propria.

An OMM was generated as a first step to serve as a basis for the HNSCC model. In line with other studies, we were able to show that primary keratinocytes and fibroblasts were suitable for the generation of a reproducible OMM [36,37,38]. As in the oral mucosa, we detected a keratinized stratified squamous epithelium in the OMM, which was separated from the lamina propria by a continuously formed basal membrane. IHC for the basal membrane marker collagen IV stained the entire lamina propria. This was most likely due to the natural occurrence of collagen IV in the SIS/MUC [39]. However, a continuous basal membrane was clearly visible by IHC. This, as well as the regular distribution of fibroblasts within the lamina propria, indicate the bio-similarity of the OMM. In addition to a suitable cell carrier, the presence of fibroblasts is particularly important in the establishment of an OMM and HNSCC model. For example, fibroblasts were shown to be significantly involved in the deposition of bioactive molecules within the ECM and in the formation of a basal membrane [40]. In the OMM, fibroblasts were evenly distributed and arranged. Interestingly, this was not the case in the HNSCC model, indicating cross-talk between cancer cells and fibroblasts. In addition to directly promoting tumor cell motility [41], fibroblasts can further support tumor cell invasion by modifying the ECM [42]. Aptly, we showed tumor cell penetration of the basal membrane with invasion into the lamina propria in our HNSCC model, generated by application of a cell mixture of FaDu cells and primary keratinocytes. In contrast, other models of oral dysplasia [43] and squamous cell carcinoma [44] failed to demonstrate this feature. Therefore, our HNSCC model succeeds in mimicking these crucial processes of HNSCC biology. We further showed the formation of tumor cell clusters with an increased number of Ki67 and p53 cells, indicating ongoing cancer cell proliferation. Cancer cell distribution and epithelial differentiation were dependent upon the ratio of FaDu tumor cells to keratinocytes. In our opinion, the optimal ratio seems to be 1:33, as tumor cell clusters with basal membrane penetration can be detected along with segments of regular epithelial differentiation. This was not the case for the other ratios used in this study.

The integration of multicellular tumor spots and tumor spheroids into our OMM on day five of ALI culture did not succeed. However, Colley et al. were able to establish an oral carcinoma in situ model by applying FaDu spheroids onto a 3D OMM generated from normal oral keratinocytes and fibroblasts transferred onto a de-epidermized acellular dermis [38]. Compared to our study, Colley et al. had seeded spheroids on their tissue-engineered OMM already before raising the model to an ALI. In our OMM, a stratum corneum and, thus, a horny layer developed shortly after raising the model to an ALI. Therefore, it is likely that the tumor cells were not able to penetrate this horny layer, thus leading to a nutritional deprivation and cell death. This discrepancy indicates that tumor cells and tumor cell spheroids should be seeded onto the models before a stratum corneum is formed, thus enabling their integration. A potentially optimal time point might be the transition from a submerged to an ALI culture. Comprising tissue-specific primary human keratinocytes and fibroblasts, an OMM served as the basis for our HNSCC model. Importantly, the OMM closely approximated the in vivo situation from a histological and immunohistochemical point of view, enhancing the model's biological relevance and its suitability as a basis for our HNSCC model. This model stands out by replicating the stroma through the use of a bioactive 3D cell carrier which mimics the tissue-specific ECM composition and features a basal membrane. Furthermore, our SIS/MUC-based models gain significance from the considerable preservation of extracellular matrix (ECM) proteins across the evolutionary development of porcine and human organisms [45], suggesting that our porcine-derived scaffold is apt for developing an HNSCC model. Interestingly, it even becomes possible to implant porcine scaffold-based constructs into patients following the scaffold recellularization with human cells [46]. In summary, key aspects of our model are a biosimilar composition of the ECM as well as invasive tumor cell clusters with basal membrane penetration and lamina propria infiltration between segments of regular epithelial differentiation, based on an OMM from primary human fibroblasts and keratinocytes. This model might help to enable further studies on tumor cell invasion as well as on the cellular and stromal interactions at the invasive tumor front. However, limitations need to be addressed. Our study aimed to develop a cancer model to elucidate general aspects of HNSCC pathophysiology, utilizing the hypopharyngeal cancer cell line FaDu integrated into an OMM. This approach is supported by literature that identifies commonalities in the disease process across various sites within the head and neck region. However, it is also crucial to acknowledge that squamous cell carcinomas can exhibit significant variations depending on their anatomical origin [47,48]. Therefore, there is a need for subsequent studies to validate these findings using further cancer cell lines from other locations of the head and neck, aiming to enhance the translatability of the results to HNSCC. Our HNSCC model does not necessarily correspond to the in vivo conditions within the TME, as it lacks key features of the natural environment. Achieving full biological relevance is difficult due to the inherent complexity of the in vivo TME, containing diverse cell types and extracellular matrix components. Our model lacks important cell types of the TME such as inflammatory and further mesenchymal cells. However, as already demonstrated by others, additional cell types such as endothelial cells [49] can be integrated into ECM-based biological scaffolds. Cell lines that have been immortalized undergo manipulation to enable indefinite proliferation. An extended period in an in vitro setting can lead to alterations in the inherent characteristics of the cell population [50]. In addition to the integration of an immortalized cell line, as was performed in our work, further approaches such as the integration of patient-derived primary tumor explants proved to be feasible in another 3D model of head and neck cancer consisting of fibroblasts seeded on a viscose fiber fabric. In contrast to cell lines, tumor explants offer the benefit of maintaining key characteristics of the cancer lesion crucial for replicating the TME, such as leukocyte infiltration [51]. Isolating primasry tumor cells directly from patient samples would better replicate patient-specific conditions, potentially increasing the model's relevance in clinical settings. In the future, such approaches might enable testing treatment responses to various anticancer drugs, assessing the tumor’s sensitivity to radiotherapy, or correlating the models to clinical histopathological characteristics like invasion and tumor growth. All in vitro models are exposed to certain microenvironmental factors, such as gradients of oxygen, nutrients, signaling molecules, and biophysical stimuli. The use of patient-derived serum, rather than calf serum, in the model could better mimic the natural tumor microenvironment, thereby providing more physiologically relevant data. However, these factors can only be controlled partially and do not exactly represent the in vivo environment of human tumors. Future efforts in HNSCC model development should focus on addressing these limitations by advancing models that incorporate primary HNSCC tumor tissue alongside various non-malignant cell types, aiming for a more comprehensive representation of the TME. By bridging the gap between basic in vitro conditions and the in vivo TME, such a model could additionally contribute to TME research and ultimately serve as platform for anti-cancer drug testing.

## 5. Conclusions

Primary human fibroblasts and keratinocytes seeded onto a porcine SIS/MUC formed a bioequivalent OMM. Cancer cells could be integrated into this OMM to generate an HNSCC model, which showed features of tumor cell invasiveness. Future work should focus on the integration of further components of the TME.

## Figures and Tables

**Table 1:** Comparison of the epithelial characteristics and tumor formation observed from the different seeding approaches.

		Oral Mucosa Model	Tumor Formation
Tumor spots		Regularly formed epithelium	No signs of malignancy or tumor formation
Tumor spheroids		Regularly formed epithelium	Tumor cells located on top of stratum corneum
Single tumor cells	1:50	Continuously formed stratum corneum, cells in the basal layers appeared irregular with nuclear pleomorphism and differences in size	Formation of tumor cell clusters; no invasion into lamina propria
	1:33	Disturbed epithelial architecture	Invasive tumor growth
	1:25	No regular epithelial structure, atypical cornification	Invasive tumor growth
	1:10	No regular epithelial structure, atypical cornification	Invasive tumor growth

## Data Availability

All data generated or analyzed during this study are included in this article. Further inquiries can be directed to the corresponding author.
